# Psychoprophylaxis, art therapy, occupational and play therapy in neuropsychiatrical recovery and rehabilitation center

**DOI:** 10.1192/j.eurpsy.2025.1287

**Published:** 2025-08-26

**Authors:** E. Chirila

**Affiliations:** 1 NEUROPSYCHIATRICAL RECOVERY AND REHABILITATION CENTER, CLUJ COUNTY COUNCIL GENERAL DIRECTORATE OF SOCIAL ASSISTANCE AND CHILD PROTECTION COUNTY COMMUNITY CENTER, JUCU, Romania

## Abstract

**Introduction:**

Many beneficiaries are stuck and find it difficult to act creatively because they are used to being in a different place than where they are at that moment:

they lose their balance and the ability to move in harmony with the forces of the environment which, in the end, will support the expression.

They expect something different from what they are doing at that moment or they think that what they are doing is inadequate; they get stuck, they get caught in negative and confused thoughts.

**Figure:**
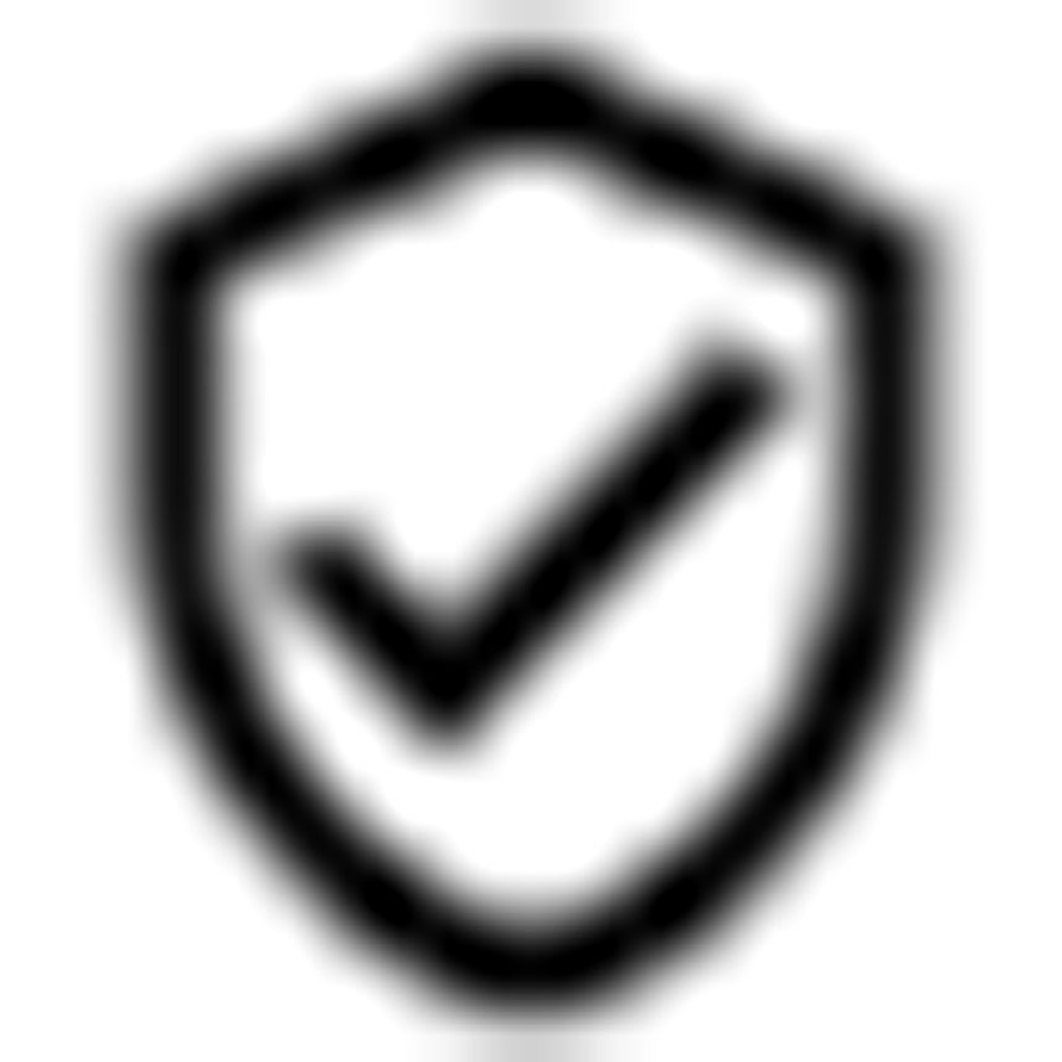

**Objectives:**

Even if a finished object or image is obtained, the research pursues, first of all, the way and the extent to which the creative process and execution of the artifact contributes to the improvement or healing of the studied beneficiaries. Correlative objective being the increase of their quality of life and their socio-professional integration. In assessing the motor functions of the beneficiaries, it is essential that the emphasis is placed on the outstanding and hidden motor abilities of each individual. The aim is to improve: coordination disorders, spatialization disorders, fine motor skills, sensoriality, creativity, socialization.

**Methods:**

ORGANIZATION OF ACTIVITIES is done in three types of workshops, with different objectives and interventions,

- Occupational therapy workshop in the category that includes art therapy.
- Occupational therapy
- Relaxation, play therapy

CASE STUDY: Placement center Jucu PA, sex M, 16 years old.

Diagnosis: Secular infantile encephalopathy, behavioral disorders

- heteroaggressiveness, severe mental retardation, alalia.
- Uses modeling for self-expression and self-fulfillment. During work, he is not aggressive and relates to people who appreciate his work.

An advanced level is observed in modeling, even compared to normal people. Spatial abstract compositions from plasticine, expressive and balanced, are made at the beginning, the evolution is observed in the modeling of the figures (he starts to model personalized human figures), which he pairs chromatically, groups them into dancing figures, or groups them affectively and vertically. He also showed changes in his behavior: he smiled while working, clapped and recognized us when we entered the workshop, wanted to be photographed and petted (although normally he can’t stand being touched). He showed interest in the camera and in the modeling works of other children from Placement center.

**Results:**

Work in visual arts and design therapies aims to create a product. The benefits arise from experiences based on artistic creativity: materializing ideas and solving unexpected results.

**Image 1:**

**Figure:**
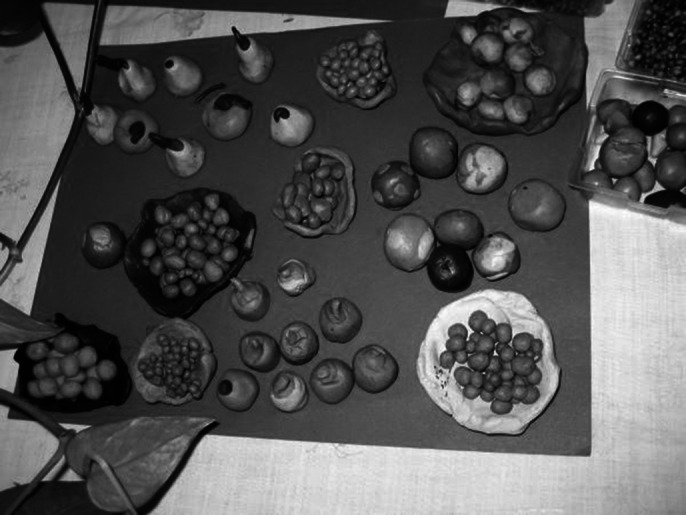

**Image 2:**

**Figure:**
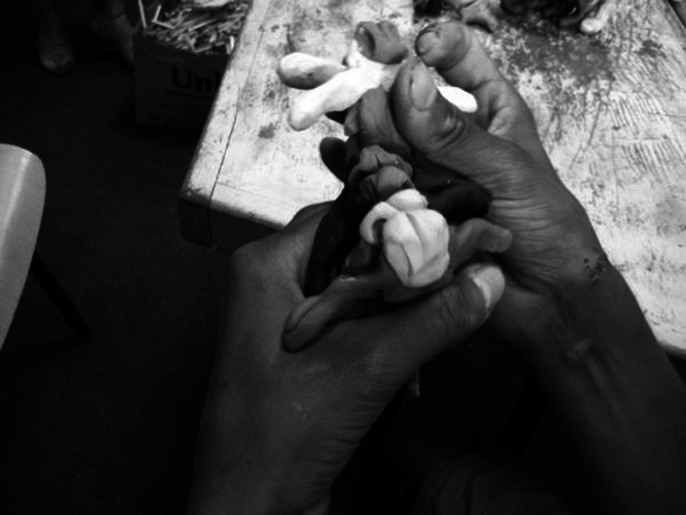

**Image 3:**

**Figure:**
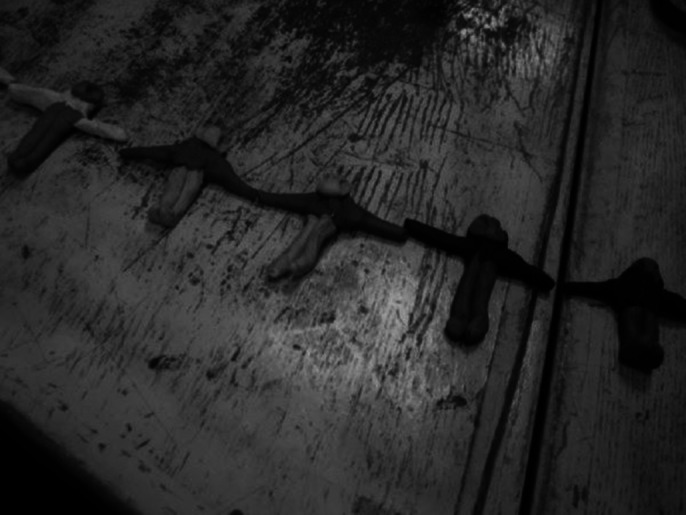

**Conclusions:**

The artistic image becomes significant in strengthening the verbal exchange between the beneficiary and the art therapist, solving problems and formulating new perceptions that in turn lead to positive changes, growth and healing.

**Disclosure of Interest:**

None Declared

